# Elimination of Defects in Mammograms Caused by a Malfunction of the Device Matrix

**DOI:** 10.3390/jimaging8050128

**Published:** 2022-05-02

**Authors:** Dmitrii Tumakov, Zufar Kayumov, Alisher Zhumaniezov, Dmitry Chikrin, Diaz Galimyanov

**Affiliations:** 1 Institute of Computational Mathematics and Information Technologies, Kazan Federal University, 420008 Kazan, Russia; kayumov.zufar@gmail.com (Z.K.); myzerix58@gmail.com (A.Z.); dmitry.kfu@yandex.ru (D.C.); 2 Medical Unit, Department of Radiology, Kazan Federal University, 420008 Kazan, Russia; da.galimyanov@mail.ru

**Keywords:** mammography, image processing, device malfunctions, interpolation, mammogram defects

## Abstract

Today, the processing and analysis of mammograms is quite an important field of medical image processing. Small defects in images can lead to false conclusions. This is especially true when the distortion occurs due to minor malfunctions in the equipment. In the present work, an algorithm for eliminating a defect is proposed, which includes a change in intensity on a mammogram and deteriorations in the contrast of individual areas. The algorithm consists of three stages. The first is the defect identification stage. The second involves improvement and equalization of the contrasts of different parts of the image outside the defect. The third involves restoration of the defect area via a combination of interpolation and an artificial neural network. The mammogram obtained as a result of applying the algorithm shows significantly better image quality and does not contain distortions caused by changes in brightness of the pixels. The resulting images are evaluated using Blind/Referenceless Image Spatial Quality Evaluator (BRISQUE) and Naturalness Image Quality Evaluator (NIQE) metrics. In total, 98 radiomics features are extracted from the original and obtained images, and conclusions are drawn about the minimum changes in features between the original image and the image obtained by the proposed algorithm.

## 1. Introduction

Neoplasms in the mammary gland represent the most common cancer type among women. Recently, the number of new patients diagnosed with breast cancer reached approximately two million per year [1,2]. The early diagnosis of lesions of the mammary gland provides good opportunities for the elimination of these abnormalities. Therefore, medical organizations around the world are trying to provide frequent and easily accessible mammography services [3].

Both mobile mammography and stationary mammography are widely used, providing different levels of detail and image quality. Mobile mammography stations often produce low-quality images. A previous paper [4] compared the image quality levels between stationary and mobile services and concluded that the quality of images from mobile devices is insufficient. Stationary and mobile devices, due to their high workload, may experience disturbances in the imaging matrix or other defects that affect the image quality. False-positive results increase the overall medical costs for society, while false-negative results do not allow the early detection of diseases.

Due to their popularity, mobile mammography services should at least provide images of acceptable quality, and the image quality should have a minimal impact on cancer detection. Due to the radiation received, it is highly undesirable to take “extra” mammographic images [5]. However, image defects are often not critical and specialists can correctly interpret the mammograms. Despite this, when conducting automatic analyses, the attained quality is not sufficient.

Image quality is especially important in automatic analysis for the correct characterization of features [6] and for the accurate operation of artificial neural networks [7,8,9,10]. It should also be taken into account that for the elimination of image defects, algorithms are used that take up a significant part of the diagnostic time.

Various approaches are used to improve mammogram images. For example, in [11], a method based on a non-subsampled shearlet transform was used to eliminate image noise. In [12], the linearly quantile separated histogram equalization grey relational analysis method was presented. This method enhanced the local and global contrast, highlighting the desired areas. In [13], several methods for eliminating noise were proposed, and the median filter, max and min filters, and Weiner filter were considered.

Dynamic unsharp masking in a Laplacian pyramid was proposed in [14] to improve the image and to better highlight the internal structure of the mammogram. In [15], a comparison of different methods of cancer detection on mammogram images was carried out. It was proposed the use of the following procedures to improve the quality: low-pass filter, Gaussian smoothing, subsampling operations, and morphological operations. To eliminate noise, median filtering was also used with a 3 by 3 window [16,17]. In [18], various image quality improvement algorithms were presented, such as the synthetic minority over-sampling technique to improve the training set; a Gaussian pyramid for image scaling with minimal loss; histogram equalization, an adaptive mean, median filters, log transforms, and a Wiener filter to increase contrast; and thresholding of the pixel intensity to eliminate artifacts. In [19], an image filtering method based on the use of a fractal mask was presented.

The most commonly used method is contrast-limited adaptive histogram equalization (CLAHE) in combination with certain other algorithms. For example, in [20], to increase the image contrast, CLAHE was used with entropy-based intuitionistic fuzzy. In [21], the image was first converted to negative and then equalization was applied. In [22], CLAHE was combined with a discrete wavelet transform to increase the image contrast. In [23], several methods of image enhancement were compared: the combination of CLAHE with bilateral filtering, a log transform, histogram equalization, a Gaussian filter, a Laplacian filter, and median filters.

In the present work, we consider the processing of mammograms, the images of which have a clearly defined defective strip with a strong increase in brightness, up to the complete illumination of the image in the area of the defect. Similar defects can occur for detectors that are in motion. As an example, normal ray detection is carried out first, which is followed by a “hardware failure” expressed in the form of a band that differs significantly in characteristics from a “good” image. Then, further detection is carried out with violations, which can cause both hardware and software distortions. These distortions are expressed in violation of the contrast or the appearance of noise and interference. In the present work, violations that are visible as blurring of the image are considered. Existing noise elimination algorithms cannot cope with such a “complex” defect. Simultaneously with the defect, there is also a decrease in the contrast of one-half of the image. An algorithm for restoring areas with a non-linear increase or decrease in image brightness is proposed. In the area of the defect, where there is a complete loss of information, the image is interpolated and restored by an artificial neural network. The reconstructed mammogram is obtained by overlaying two images, which are interpolated (information about the light lines on the mammogram) and reconstructed by a neural network (information about the background).

The processed image should have approximately the same contrast; the vessels, fibrous and glandular tissues contained in the image should be well “read”. We use the BRISQUE and NIQE metrics to assess the perception of the entire image, as well as ten other metrics to assess the accuracy of recovering a lost image (an image in the band area). A comparison is also made on 98 radiomics features extracted from the original and obtained images.

## 2. Materials and Methods

Mammogram images contain artefacts and may have low contrast, which can significantly complicate the diagnosis process. The image recovery algorithm contains the following steps:

- Determining the core of the defect (black pixels and excessively bright pixels);
- Defect determination. Restoration of pixels outside the core;
- Equalizing the contrast of the entire image, except for the defect;
- Restoration of the core of the defect by the interpolation algorithm;
- Selection of light lines on the restored image;
- Restoration of the background of the core of the defect and of the area adjacent to the core with an artificial neural network;
- Overlaying of the interpolation results (highlighted lines) on the background obtained by the neural network.

Next, we take a closer look at some of the algorithms used.

### 2.1. Improving the Image Contrast

One of the main tasks in image processing is to increase the contrast. Contrast creates a visual difference that distinguishes an object from the background and other objects. The main goal is to improve the visual quality of the image. Histogram equalization (HE) is a commonly used method for modifying a histogram. However, the HE is a global image adjustment method that cannot effectively improve the local contrast, as the effect will be very poor in some situations. Therefore, the contrast-limited adaptive histogram equalization (CLAHE) method is used.

In the present work, we use the CLAHE algorithm to remove noise and enhance the contrast of mammograms. The filter parameters directly depend on the modes of intensity distributions on opposite sides of the defect. For the function in python, we choose grid_size = 8 by default, while clip_limit is set to values on a case-by-case basis. Other contrast enhancement algorithms can also be used, for example the multi-fractal method [16].

### 2.2. Recovery of the Lost Image

The classic approach for recovering a lost image is interpolation or approximation. Algorithms based on the Fourier spectrum, performing phase, and amplitude reconstruction are also used [24]. The main idea of this method is the sequential calculation of the image spectrum, with changes made only to the defect without changing the rest of the image [25,26,27].

In recent years, artificial neural networks have also been widely used [28,29]. The main advantage of the method is the high speed of work on high-resolution images with an acceptable level of restoration quality and the simplicity of the training set.

In the present work, two neural networks are used for the algorithm: a coarse–fine network for coarse gap filling in an image compressed to 256 × 256 and a refine network for more accurate gap filling in an image compressed to 512 × 512.

To improve the accuracy of filling a gap in the original image, the following parameters are calculated:

- The contextual residual, which is the difference between an original image and an image obtained after downsampling to 512 × 512 and further upsampling to an original resolution;
- Attention scores, which act as characteristics of the region affinity of the part of the image outside the gap to the gap filled by the neural network. They are used to transfer image structure information outside the gap to the inside.

Based on these two parameters, aggregated residuals are calculated, which are then added to the filled gap for sharpening.

### 2.3. Image Binarization

Binarization is one of the effective ways to determine the threshold level of shades of gray for stratification of the original image into the analyzed object and background. The quality of the image binarization process is improved as a necessary condition for increasing the efficiency of selection (detection) of given objects [30]. The binarization threshold is defined as an optimization problem in determining the maximum measure of distinguishability of classes [31] or is chosen empirically. Binarization is also actively used in mammography to eliminate image noise [32,33]. Other approaches are also used to highlight an image against the background; for example, the thresholding method and morphological operations were effectively applied in [17].

The main parameter of such a transformation is the threshold, which represents a value with which the brightness of each pixel is compared. Various binarization methods exist that can be conditionally divided into two groups: global and local. In the former case, the threshold value remains unchanged during the entire binarization process. In the latter case, the image is divided into regions, in each of which a local threshold is calculated. There also exist methods (for example the Otsu method) in which a threshold is automatically calculated that minimizes the average segmentation error, i.e., the average error from deciding whether image pixels belong to an object or background.

The main goal of binarization is a radical reduction in the amount of information that one has to work with. In the present work, incomplete thresholding with a global threshold (determined empirically) is used to extract light areas in the image.

## 3. Results

**Figure 1:**
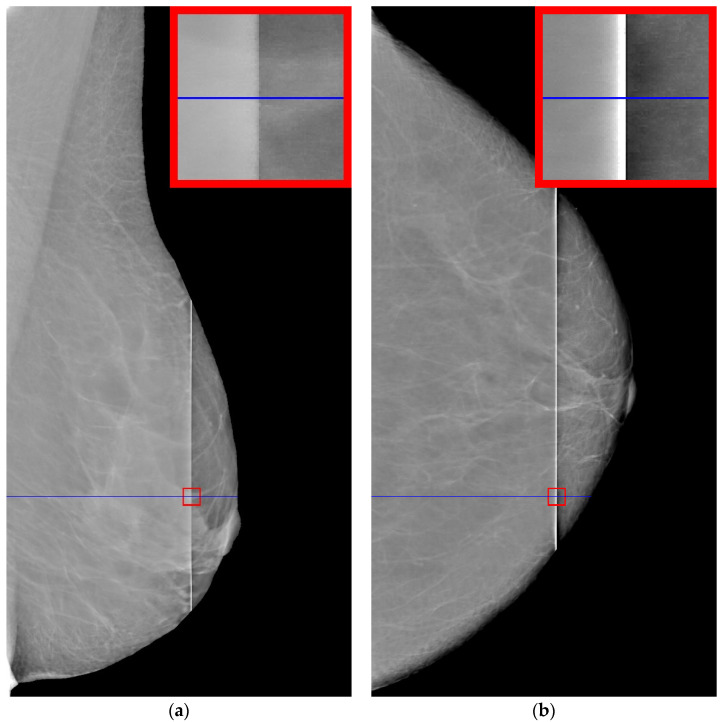
Examples of defective mammograms. The size of the red squares is 100 by 100: (**a**) mammogram with unilluminated defect; (**b**) mammogram with illuminated defect.

The original image of 4052 by 5539 pixels contains a vertical defect, which consists of a sharp increase in the intensity of the image in the area of the defect. The defect may have a different width along the vertical line. Defect values can reach from several tens to hundreds of pixels. The increase in brightness in the defect strip can be quite significant, in some cases reaching full illumination (maximum brightness). Examples of such mammograms are shown in Figure 1. The mammograms are taken retrospectively from patient records.

**Figure 2:**
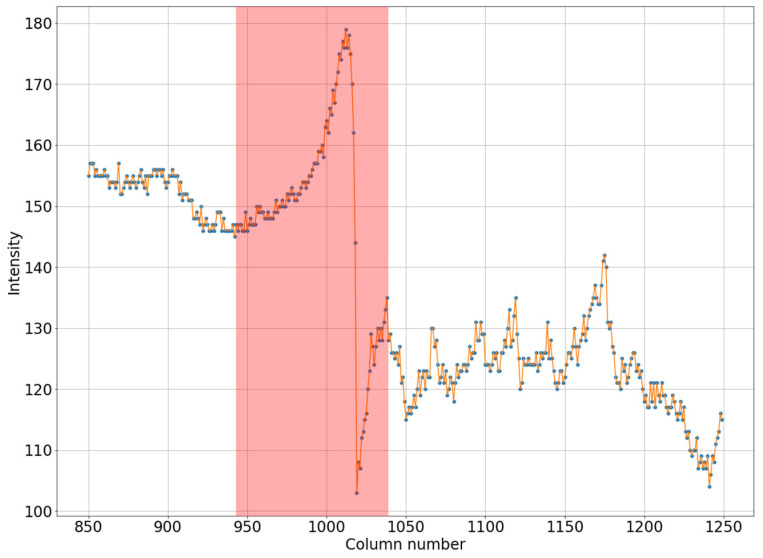
Horizontal section of a mammogram with a wide low-intensity defect which corresponds to the blue stripe in Figure 1a.

**Figure 3:**
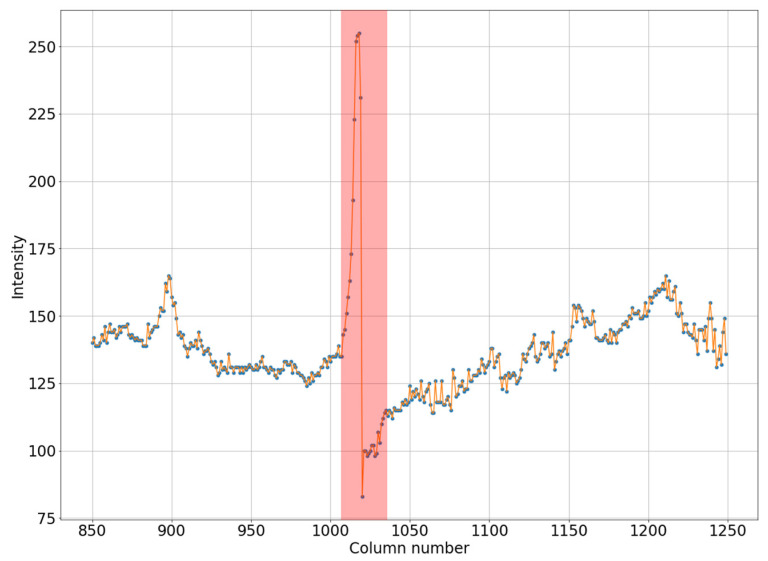
Horizontal section of a mammogram with a narrow high-intensity defect, which corresponds to the blue stripe in Figure 1b.

It can be seen in Figure 1a that the intensity does not reach maximum values in the region of the defect. However, the area of increased brightness is quite large. In Figure 1b, there is an illuminated defect, and in this area there is a complete loss of information. Unlike images in which the brightness increase is slow, in this case the brightness increase is quite sharp. This can be clearly seen in Figure 2 and Figure 3.

Let us note the following. A defect always shows an increase in pixel intensity on one side with a simultaneous decrease in intensity on the other side of the defect. In addition, the width of the defect correlates with its intensity. A wide defect corresponds to low-intensity changes. Conversely, a narrow defect corresponds to high-intensity distortions. For the proposed algorithm, it is necessary that one of the areas have an image with high contrast, corresponding to an operational device.

Let us consider the results of the step-by-step reconstruction of the mammogram image with illustrations using the example shown in Figure 1b.

### 3.1. Determination of Defects

At the initial stage, we determine the contour of the chest. This operation can be performed by moving from the borders of the photo to the center until the pixel color stops being black or reaches a specified low value. Next, we find the core of the defect. We consider the core of the defect to be the part of the image where the intensity is more than 220 or equal to zero. Note that there may not be a defect core in a particular horizontal section. Let us explain this with the example of Figure 1a. Here, you can see that the brightness of the strip in the middle is smaller than in the upper and lower parts. Thus, in this case, the defect core will be only at the ends of the vertical defect. In the case when the core of the defect is not connected, we will connect the individual sections with straight lines, resulting in a vertically similar area.

**Figure 4:**
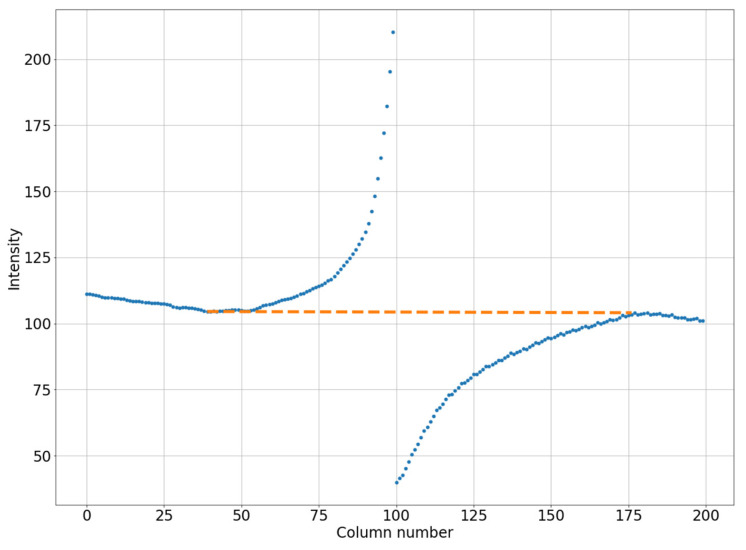
An example of linearization of the column-average intensity in a small square (200 by 100 pixels). The blue dotted line is for the original image. The orange dotted line is for the smoothed image.

In the next step, the areas to the left and right of the defect core will be covered with curvilinear quadrilaterals measuring 100 × 100 pixels in size (in practice, this area will have an arcuate shape along the vertical). Then, we will calculate the average pixel intensities for each column of the selected quadrilateral. Let us determine the minimum intensity value from the illuminated side and the maximum intensity value from the darkened side, then connect the obtained data with a straight line, as shown in Figure 4. We determine the difference between the average values in each column (blue line in Figure 4) and the straight line values (orange line in Figure 4). The resulting value is calculated from the intensity values for each point of the column, thereby adjusting the brightness of the pixels in the defect area.

Let us repeat this action for all columns of the rectangle. Apparently, as the distance from the defect core increases, the change in the intensity values decreases in each column of this area, correcting the average pixel values to the desired level. The outer borders of the upper and lower rectangles coincide with the contour of the chest, and their vertical size can be less than 100 pixels.

In the next step, we will remove the “blurring” of the image of the area with low contrast.

### 3.2. Equalizing Contrast in the Image

**Figure 5:**
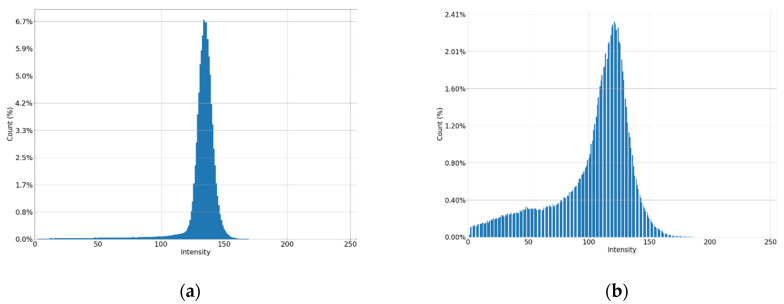
Histograms of parts of the image: (**a**) low contrast image; (**b**) high contrast image.

Let us estimate the contrast of the image to the left and to the right of the defect. The blurrier (lower contrast) image shown in Figure 5a corresponds to mammogram shown in Figure 1b. A high-contrast “uncorrupted” image of the same mammogram is shown in Figure 5b.

Let us improve the “corrupted” image. For this purpose, we use the CLAHE method with the clip_limit parameter, which must be determined by the ratio of low- and high-contrast images. We calculate the mode and standard deviation (stdA and stdB) for the distribution of pixel brightness on both sides of the defect. We determine the CLAHE parameter using the following formula:

clip_limit = 1.11 − 0.023 × (stdB − stdA)(1)

**Figure 6:**
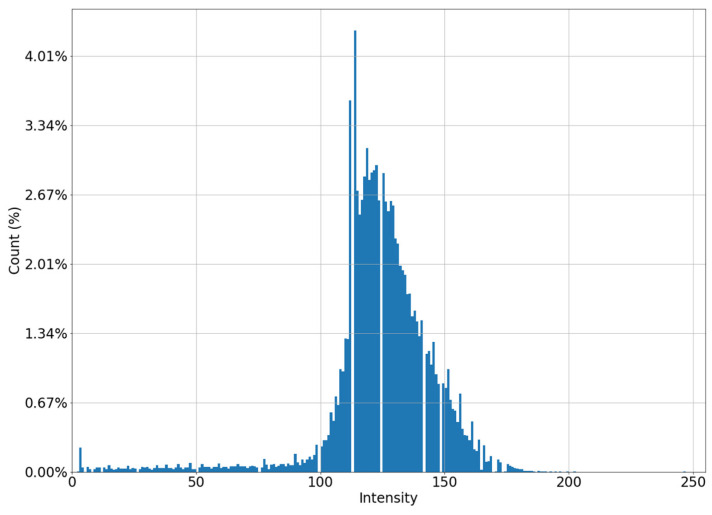
Histogram of a “low-contrast” image after applying the CLAHE algorithm.

This formula is empirically derived as a regression model built on optimal clip_limit values for multiple mammograms. As a result, we improve the image for the non-contrasting part by changing the histogram from Figure 5a to Figure 6.

After this stage, the entire image, except for the core of the defect, has approximately the same contrast. Now let us proceed directly to the restoration of the image inside the core of the defect.

### 3.3. Restoring the Image in the Core of the Defect

**Figure 7:**
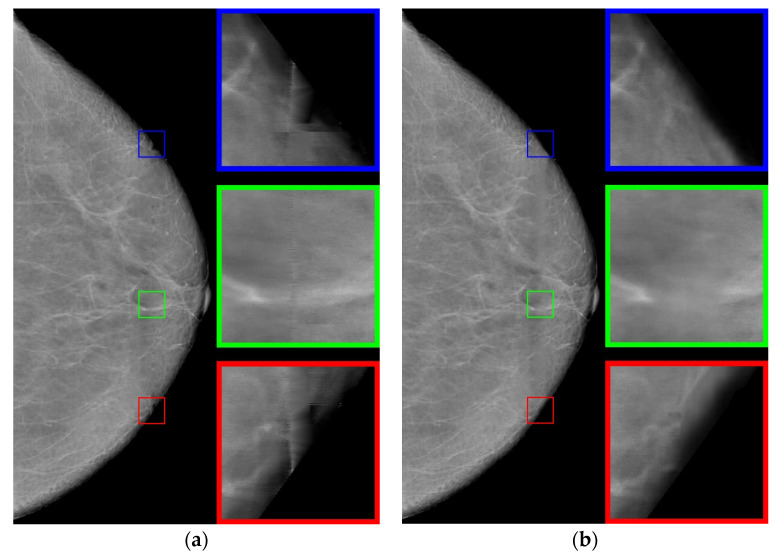
Restored images: (**a**) using the interpolation method; (**b**) using the neural network.

Using the interpolation method, we restore the images in the strip. The use of polynomials of higher degrees gives artifacts to the image; therefore, we use polynomials of the first degree. The resulting image is shown in Figure 7a. Note that the use of polynomials of the second or third degree does not significantly change the result.

Let us analyze enlarged images from small squares. It can be seen that the edge images (blue and red squares) are poorly restored. The images inside are approximated well enough, retaining the “white streaks”. However, with this approach, there are small horizontal stripes (green square), which is a usual occurrence with approximation by polynomials of a small degree.

Now we apply an artificial neural network trained on other data [29], based on the architecture of generative adversarial networks (GANs), which are very often used to generate natural images and videos. The resulting image is shown in Figure 7b. For the neural networks, the image is well restored at the boundaries (blue and red squares). However, the image inside the defect is blurred, nevertheless preserving the overall background well.

**Figure 8:**
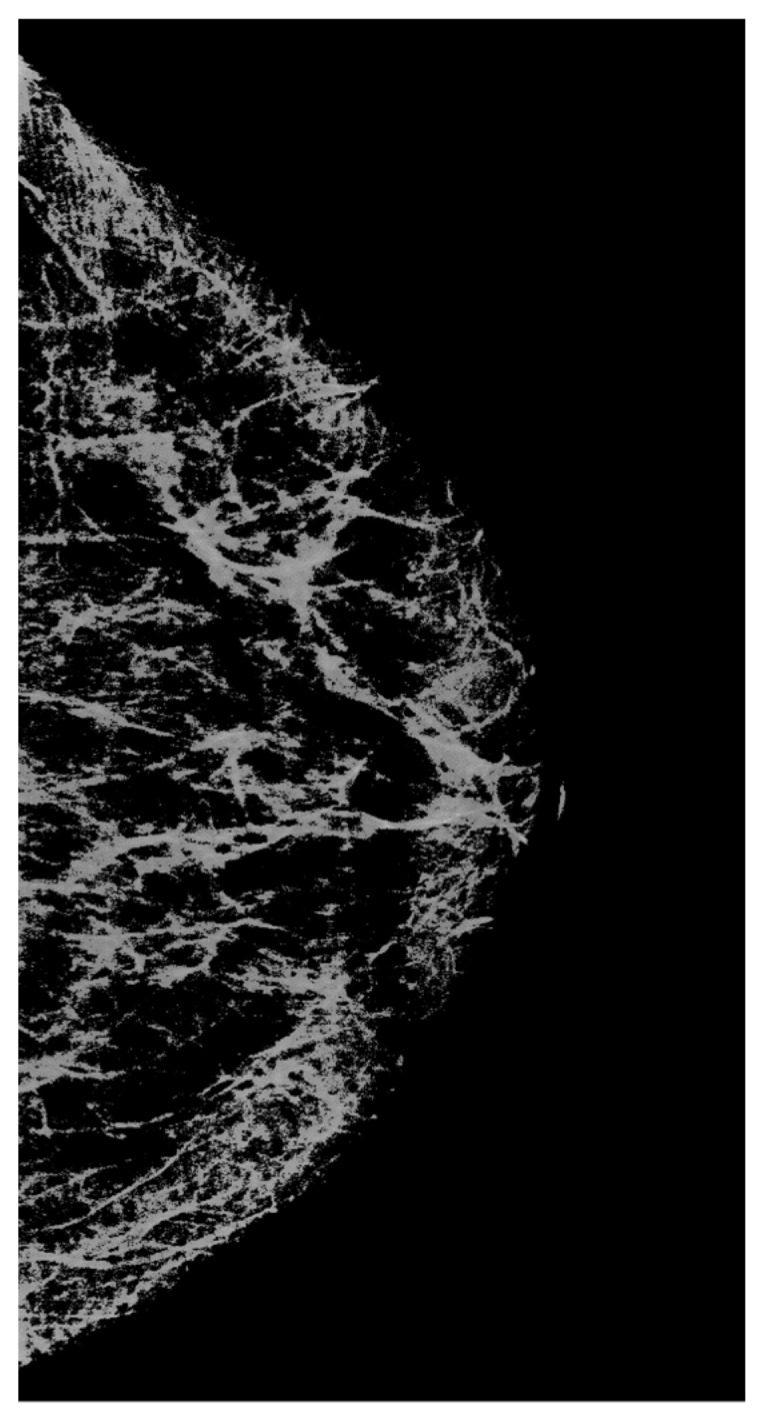
A mask that carries information about significant lines in the image.

Let us proceed as follows. Let us take white streaks from the image restored by interpolation (defect pattern) and transfer them to the image obtained by the neural network (defect background). To do this, we apply the CLAHE method with the clip_limit = 20 parameter, significantly increasing the contrast. Then, we carry out binarizations. We attribute to the background the pixels with intensity levels of less than 2/3 of the maximum brightness (= 170), whereas the pixels with intensity levels ranging from 171 to 255 are attributed to the informative part of the image. We obtain a binary mask (Figure 8).

**Figure 9:**
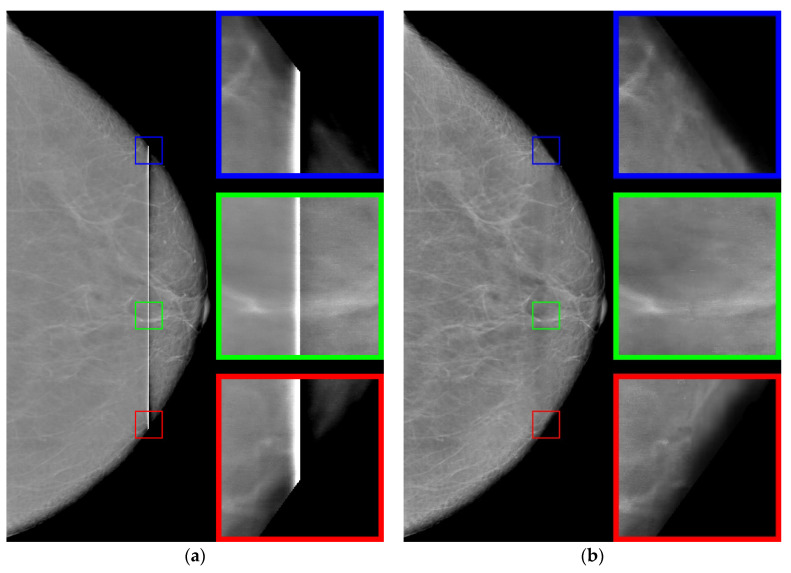
(**a**) The original image and (**b**) the restored image.

Using the mask, we select the necessary data (from Figure 7a) and apply them to the image containing the background (Figure 7b). As a result, we obtain the image shown in Figure 9b.

### 3.4. Comparison of the Algorithm Versus Other Methods

**Figure 10:**
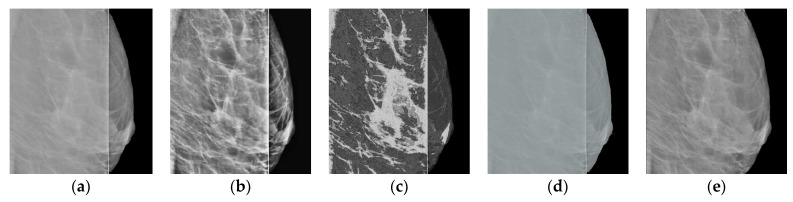
Results of processing shown in Figure 1a: (**a**) original image; (**b**) CLAHE; (**c**) MedGA; (**d**) SCL-LLE; (**e**) proposed method.

**Figure 11:**
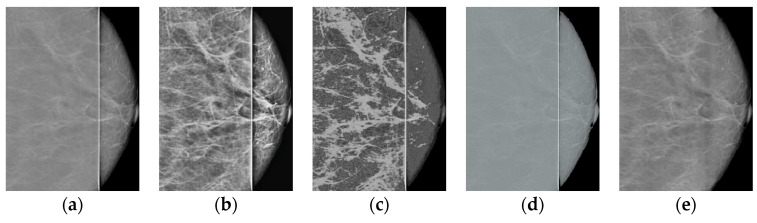
Comparison of results for Figure 1b: (**a**) original image; (**b**) CLAHE; (**c**) MedGA; (**d**) SCL-LLE; (**e**) proposed method.

A comparison of the results obtained with the proposed algorithm versus the results obtained with existing methods for improving the image quality is shown in Table 1. HERE, The most common metrics for measuring image quality without a reference, namely BRISQUE [34] and NIQE [35], Are used. It can be seen that the method proposed in the present work achieves the best value for the considered images. The following methods Are used for comparison: CLAHE with the clip_limit = 5 parameters; MedGA medical image enhancement method [36] based on genetic algorithms; neural network SCL-LLE [37] to improve the quality of the input image. The results of processing shown in Figure 1 for these methods are presented in Figure 10 and Figure 11.

Thus, the proposed method in terms of BRISQUE and NIQE metrics achieves the best value for the images considered in the work. Additionally, note that the MedGA and SCL-LLE methods do not remove the vertical defective band.

### 3.5. Estimating the Accuracy of Recovering a Lost Image

**Figure 12:**
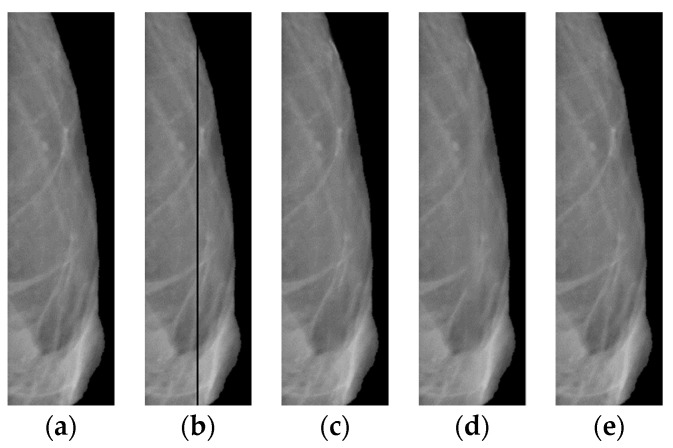
Right side of the image shown in Figure 1a: (**a**) original; (**b**) after cutting out the rectangular strip; (**c**) with interpolated band; (**d**) with a strip reconstructed by a neural network; (**e**) with the strip recovered by the proposed algorithm.

Let us evaluate the algorithm used for recovering a lost image. We use for this purpose the “good” (right) part of the mammogram image from Figure 1a. On the original image (Figure 12a), we cut out a rectangular strip 5 pixels wide (see Figure 12b). First, we restore the lost image in the strip using the classical interpolation method; the results of the restoration are shown in (Figure 12c). Then, we apply an algorithm based on a neural network to the defective image (Figure 12b).

The following reference metrics are used here to assess the overlay efficiency: SSIM [38], VIF [39], VSI [40], CW_SSIM [41], MS_SSIM [42], FSIM [43], DISTS [44], GMSD [45], LPIPS-VGG [46], and NLPD [47]. The results for all metrics (Table 2) show that after the overlay, the image is better in comparison with the separate results for the application of the neural network and interpolation processes.

### 3.6. Radiomics and Statistical Analyses

Let us evaluate the features of four groups of images, the original image and three transformed images obtained by the algorithms discussed above, namely the proposed method, MedGA, and SCL-LLE. These evaluations will be carried out using Radiomics, which is used to extract a large number of features in medical images. Radiomics is also frequently used in the detection of various visually detectable diseases and abnormalities [48,49]. In the present work, the pyRadiomics python package [50] is used for feature calculations.

The Radiomics values not only indicate the quantitative histogram and texture characteristics of a medical image, but are also used as input data for machine learning [51,52,53]. Therefore, it is important to evaluate these parameters in the converted images in order to assess the degree of change in the original image. Since the method proposed in the work has significantly improved the perception of the picture, it is also necessary that the characteristic features not be “spoiled” in this case.

Let us single out 93 features: 18 for first-order statistical descriptors and 75 for texture features. Then, we can compare the obtained values using one-way analysis of variance (ANOVA) and Tukey’s post hoc honestly significant difference (HSD) test to identify very different features [49]. ANOVA is used here to determine whether there are any statistically significant differences between the group for the original images and the processed images. In other words, one-way analysis of variance compares the means among groups (in our case, four groups), determining whether one of these means is statistically different from the others.

**Figure 13:**
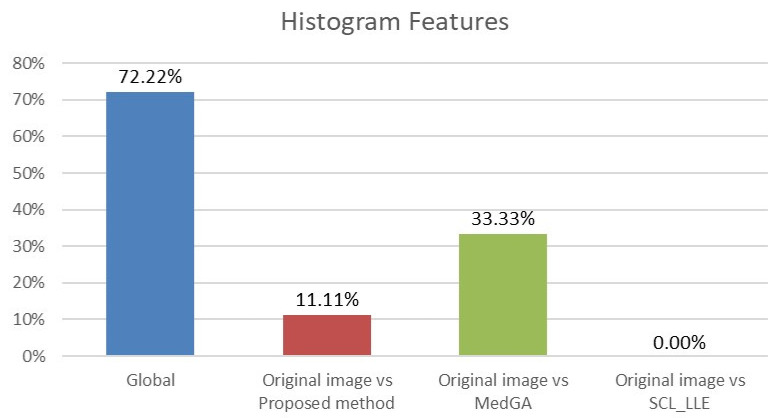
Analysis of ANOVA and HSD tests showing differences in histogram features (18 features) across four groups (blue column) and differences considering pairs of groups: first group vs. second group (red column), first group vs. third group (green column), and first group vs. fourth group (purple column).

**Figure 14:**
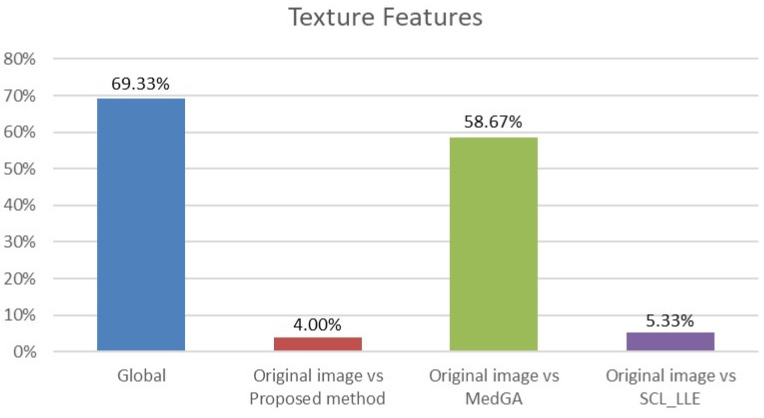
Analysis of ANOVA and HSD tests showing differences in texture features (75 features) across four groups (blue column) and differences across pairs of groups: first group vs. second group (red column), first group vs. third group (green column), and first group vs. fourth group (purple column).

Specifically, it tests the null hypothesis:

$$H_{0}=\mu_{1}=\mu_{2}=\cdots =\mu_{5}\;$$

where $\mu_{k}$ is the group mean and k is the group number. If the ANOVA returns a statistically significant result (*p*-value below 0.05), we accept the alternative hypothesis that there are at least two group means that are statistically significantly different from each other. We then look at radiomics features with *p* < 0.05 in a one-way ANOVA to find the percentage of radiomics features that differ between at least two groups. The ANOVA does not provide information about which group is significantly different from the others, but only that at least two groups are statistically different. For this reason, the Tukey HSD test must be used. This is a post hoc test based on Student’s t-distribution, which is useful for determining which of the group pairs are significantly different from each other. A parameter for each pair, namely the Tukey HSD Q-statistic, is calculated as follows:

$$Q-\mathrm{statistic}=\frac{{\bar{X}}_{i}-{\bar{X}}_{j}}{\sqrt{\sigma_{k}^{2}/n}}\;$$

where ${\bar{X}}_{i}$ and ${\bar{X}}_{j}$ are average values of the compared samples; *n* is the sample size; $\sigma_{k}^{2}$ is the within-group dispersion. Then, the *p*-value of the comparison of the observed Q-statistic and the Q-critical one is calculated. Finally, radiomics features with Tukey HSD *p*-values < 0.05 are examined to determine the percentage of features that differ between the original image and the considered approaches. This makes it clear which of the approaches shows a statistically significant difference. The results for histogram features (First-order features) and for texture features are shown in Figure 13 and Figure 14.

Analyzing the data in the histograms, we can conclude that the method proposed in this work leaves practically all of the signs without significant changes, except for 5 signs. For histogram features, these are the maximum intensity value and the range of intensity spread. For textural features, these are the gray-level size zone matrix (GLSZM) features: short run emphasis (SRE), size-zone non-uniformity normalized (SZNN), and small area emphasis (SAE) features.

## 4. Discussion

The problem solved in the present study is purely practical. The defect is not significant enough to justify purchasing new mammographs; however, it cannot be technically eliminated. The proposed algorithm improves the images, making them acceptable for analysis by a specialist or for further computer processing.

In the algorithm, when restoring the background of a defect, a neural network trained on third-party data (not mammograms) can be used, since this is sufficient to restore the background. Table 2 shows scores on ten metrics for three algorithms for recovering a lost image. For all metrics, the combined use of interpolation and a neural network gives the best results. Additionally, note that the algorithm can be easily applied to mammograms of any size.

If assessed visually without zooming in, the mammogram reconstructed by the interpolation algorithm (Figure 7a) looks quite contrasty. However, when zoomed in (colored squares in Figure 7a), it becomes clear that this is definitely not enough, and further improvement of the image is needed. A comparison of the proposed algorithm versus other approaches in terms of BRISQUE and NIQE metrics (Table 1) indicates that it provides better results. Adding “restoration” of the band to other approaches will lead only to an insignificant improvement in their metrics shown in Table 1, and will not change the overall score.

In total, 98 radiomics features are extracted from the original and “improved” images. Two differences in histogram features are explained by the presence of an overexposed band in the original image. If the band is excluded from consideration, then only three different textural features remain. We also note that in addition to the visual component, the MedGA method also significantly changes the features of radiomics, as indicated by the values of 33.33% and 58.67% of features that differ from the original image.

## 5. Conclusions

An algorithm for eliminating defects in a mammogram distorted by equipment is proposed. The mammogram has a defective strip in the form of a sharp change in the intensity of the pixels in this area up to a complete loss of information in certain areas of the strip. In addition, one part of the image has a lower contrast than the other part.

The algorithm combines the following steps:

- Defect highlighting;
- Equalization of image contrast outside the defect;
- Restoration of a defect by a combination of interpolation and artificial neural network.

One of the significant results of this work is an effective combination of two approaches to image restoration. One algorithm restores the background, while the other one restores the “significant” image. The results of the two algorithms are then combined to form a new high-quality image without loss of radiomics features.

## Figures and Tables

**Table 1:** Evaluation of various existing methods for improving image quality as well as of the proposed method for images from Figure 1.

Input Image	Methods	Metrics	
		BRISQUE ↓	NIQE ↓
Figure 1a	Original image	18.72	10.41
	CLAHE	24.68	47.54
	MedGA	95.44	14.03
	SCL-LLE	17.75	9.18
	Proposed method	**15.55**	**5.22**
Figure 1b	Original image	14.34	9.77
	CLAHE	40.01	10.17
	MedGA	72.83	12.21
	SCL-LLE	14.89	7.79
	Proposed method	**10.61**	**5.95**

**Table 2:** Evaluation of methods for restoring a “lost” image in a strip artificially cut out on the right side of the image shown in Figure 1a.

Metrics	Original Image	NN	Interpolation	Overlay
SSIM ↑	1.000	0.947	0.921	**0.994**
VIF ↑	0.999	0.644	0.607	**0.867**
VSI ↑	1.000	0.987	0.979	**0.998**
CW_SSIM ↑	1.000	0.928	0.869	**0.997**
MS_SSIM ↑	1.000	0.957	0.925	**0.999**
FSIM ↑	1.000	0.956	0.936	**0.995**
DISTS ↓	0.000	0.030	0.036	**0.001**
GMSD ↓	0.000	0.093	0.116	**0.023**
LPIPS-VGG ↓	0.000	0.011	0.023	**0.001**
NLPD ↓	0.000	0.855	1.087	**0.195**

## Data Availability

Data based on information that retrospectively has been taken from the patient’s case reports.
